# Clinical application of multicolor imaging in Leber hereditary optic neuropathy

**DOI:** 10.3389/fneur.2022.1003514

**Published:** 2022-10-28

**Authors:** Yufang Cheng, Lu He, Qingmei Miao, Wenyu Wang, Jiajia Yuan, Changzheng Chen

**Affiliations:** ^1^Eye Center, Renmin Hospital of Wuhan University, Wuhan, China; ^2^Physical Examination Center, Renmin Hospital of Wuhan University, Wuhan, China

**Keywords:** multicolor imaging, retinal nerve fiber, Leber hereditary optic neuropathy (LHON), multicolor confocal scanning laser ophthalmoscopy, diagnosis

## Abstract

**Purpose:**

To characterize features of retinal never fiber in Leber Hereditary Optic Neuropathy (LHON) using multicolor (MC) imaging and color fundus photography (CFP).

**Methods:**

Ninety-two eyes of patients with LHON underwent MC imaging, optic disc spectral domain optical coherence tomography (SD-OCT), and CFP. Two independent observers graded RNFL visibility scores and two other experts determined never fiber bundle defects from four-quadrant readings. CFP, standard MC, infrared reflectance (IR), green reflectance (GR), blue reflectance (BR), and green-blue-enhanced (BG) imaging were compared.

**Results:**

Agreement on never fiber bundle defects was substantial for CFP, standard MC, GR, BR, and BG images relative to IR. It was shown that BR (2.71 ± 0.55) had the best mean RNFL visibility score, BG (2.69 ± 0.52), GR (2.69 ± 0.56), standard MC (2.04 ± 0.79), CFP (1.80 ± 0.82), and IR (0.45 ± 0.59) followed. Agreement on temporal area defects was relatively improved. Youden's indices for CFP (78.21%), standard MC (84.48%), GR (90.92%), BR (89.64%), and BG (90.99%) indicated good detection of defects in the papillomacular bundle (PMB)/ high suspicion of patients with LHON, particularly for BG and GR. According to the proportion of never fiber bundle defects, standard MC, GR, BR, and BG can roughly determine the LHON clinical stage, especially in subacute and chronic stages, and standard MC is superior for patients with LHON of all stages. The stage judged by MC was consistent with the course inferred by pRNFL thickness.

**Conclusion:**

As an adjunct to SD-OCT, the MC image, particularly the GR and BG can delineate RNFL more effectively than CFP. The MC image may be a useful adjunct to OCT for detecting or monitoring never fiber bundle defects, providing inexpensive and rapid methods that can quickly identify patients with high suspicion of LHON.

## Introduction

Leber hereditary optic neuropathy (LHON) is the first disease that has been associated with mitochondrial DNA (mtDNA) point mutations (1). It is a blindness disorder, usually affecting young men, that leads to the selective degeneration of retinal ganglion cells and optic atrophy within a year of disease onset (2). Therapeutic options for LHON, including innovative drugs with different target pathways, are currently under study (3). Idebenone (Raxone^®^) (3) and gene therapy (4) have emerged as the most promising to date. Historically, LHON has been categorized into three distinct clinical groups: asymptomatic mutation carriers, patients with acute LHON (disease duration of 1 year or less), and patients with chronic LHON (disease duration of >1 year). Both idebenone and gene therapy have been shown to increase the rate of visual recovery in patients with LHON, especially when given in the acute stage (5–7). More recently, assessment by optical coherence tomography (OCT) across different stages of LHON, in conjunction with natural history and established visual parameters, such as best-corrected visual acuity (BCVA) and visual fields (VFs), has provided new insights into the clinical stages of LHON. At an international consensus conference held in Milan in 2016 (8), experts defined clinical stages of LHON, subdividing the acute stage into “subacute” and “dynamic” stages. Experts strongly agreed that the subacute and dynamic stages should be targeted for early treatment to prevent further loss of fibers and vision. Overall, the “the sooner the better” paradigm is central to clinical practice. Unfortunately, despite the greatly improved recognition of LHON and the availability of genetic testing, many patients still suffer from considerable diagnostic delay. A differential diagnosis remains an important point of consideration, particularly with regard to optic neuritis (7). Genetic testing is the gold standard for the diagnosis of LHON. However, expanding genetic testing will increase the financial burden on patients and waste resources. There is an urgent need for inexpensive and rapid methods that can quickly identify patients with high suspicion of LHON. Targeted genetic testing can be later performed on suspects, to reduce missed diagnoses and shorten the diagnostic cycle. In addition, there are certain difficulties in staging patients with LHON. In most cases, LHON is bilateral or asymmetric, with one eye losing vision first and the other following within days, weeks, or months (2). It is notoriously difficult to subdivide patients with LHON based solely on their recollections of symptom onset, since well-established cases can present only after the second eye becomes clinically involved (5).

Multicolor confocal scanning laser ophthalmoscopy (cSLO) imaging is a non-invasive retinal imaging approach, performed by Spectralis SD-OCT (Heidelberg Engineering, GmbH). It can collect images captured with monochromatic laser sources of different wavelengths. Blue reflectance (BR, λ = 488 nm), green reflectance (GR, λ = 515 nm), and infrared reflectance (IR, λ = 820 nm) have different penetration ranges and thus can be used to visualize structures at different depths within the retina (9) (GmbH HE Heidelberg Engineering Academy–SPECTRALIS MultiColor–Interpreting MultiColor images in three steps. https://academy.heidelbergengineering.com/course/view.php?id=204 accessed 20 July 2020). A standard MC image and green-blue-enhanced(BG)image are subsequently computed (9). Several studies have reported the usefulness of this approach for the detection of RNFL defects in glaucoma (10, 11), but its application to LHON has not yet been fully investigated. Thus, we investigated the RNFL visibility score and characteristics of fiber bundle defect in patients with LHON, by MC and color fundus photography (CFP) in the present study. Aiming to improve the clinical detection of suspected patients with LHON, or to quickly identify patients like LHON, preferentially involve fibers in the papillomacular bundle (PMB) area, in a non-invasive and inexpensive manner. Besides, we also explored the feasibility of judging the clinical stages of LHON based on the proportion of retained and lost fiber bundles on fundus photography.

## Methods

This study included 148 eyes from 74 participants. Age, gender, refractive value, LHON duration, treatment experience, and family history were collected. For all 74 participants, both eyes were subjected to complete ophthalmic examinations, including BCVA, intraocular pressure (IOP), slit-lamp examination, gonioscopy, 30-2 Humphrey VF (2010 Carl Zeiss Meditec HEAI740-40979-51.2/5.1.2), and dilated fundus examination. For each participant, CFP (Digital Fundus Camera, VISUCAM 200; Carl Zeiss Meditec AG, Jena, Germany), MC imaging, and optic disc spectral domain OCT (SD-OCT) (Heidelberg Engineering, Heidelberg, Germany) were performed after full dilation. Artifact-producing factors were controlled in our study (12). MC images were acquired concurrently or immediately after SD-OCT imaging. The MC scan angle was 30°, the IR laser power was 25% (powers of the blue and green lasers were not adjustable), and the automatic real-time was set to 25. Standard MC and BG images were composed of three reflective images. Pseudocolor channels, brightness, contrast, and sharpening parameters were not manually altered for the images evaluated in this study. All examinations were performed by a skilled technician. Included images contained, at minimum, the temporal edge of the optic nerve, the fovea, and the arcades nasal to the fovea. Images were excluded for low quality as judged by the authors or due to artifacts.

The inclusion criteria for symptomatic carriers were as follows: (1) age between 12 and 55 years; (2) painless visual loss in one or both eyes with central scotoma or paracentral scotoma; and (3) genetically proven pure m. *G11778A* mutation with LHON. The inclusion criteria for asymptomatic carriers were as follows: (1) mothers of symptomatic carriers with genetically proven pure *m.G 11778A* LHON mutation, mothers' siblings, or symptomatic carriers' siblings; (2) BCVA of 1.0 (0.0 LogMAR) in both eyes; and (3) no visual field defects.

Exclusion criteria were as follows: (1) IOP > 21 mmHg, closed angle on gonioscopy, presence of glaucoma, ischemic optic neuropathy, or other neuro ophthalmological diseases; (2) spherical equivalent >-6.0 diopter; (3) other ocular diseases affecting the cornea, lens, or retina; (4) severe physical illness; and (5) poor subject fixation, leading to low quality obtained images and lack of cooperation with the researcher.

### Grading and evaluation of images

Two specialists examined all of the SD-OCT optic disc data, CFP, and MC images individually on a high-quality monitor under dim lighting conditions. Specialists were all masked to the patients' clinical information. First, the specialists evaluated RNFL visibility on a 4-point scale (0 = no visibility, 1 = poor visibility, 2 = fair visibility, and 3 = excellent visibility) in a masked and independent manner. Then, two other specialists determined the grade (normal, swelling, partial defect, and serious defect) and location of the never fiber bundle defects (none, superior, temporal, inferior, nasal) independently on each of the images. Localized never fiber bundle defects were defined as wedge-shaped defects running toward the optic disc.

The determination of LHON was based on observed almost complete defects in retinal never fibers in the PMB area. Clinical stages of LHON were divided into asymptomatic (mutation carriers), subacute (<6 months from onset), dynamic (6–12 months), and chronic (>12 months) categories, based on peripapillary retinal nerve fiber layer (pRNFL) thickness. Two specialists judged the clinical stages of patients with LHON based on the proportion of never fiber bundles defects in four quadrants for each independent image. Disagreements were resolved by consensus. The sensitivity and specificity of never fiber bundle defects detection were determined based on the graders' overall diagnoses.

### Statistical analysis

No significant never fiber bundle defects were found in the asymptomatic carriers. While specialists read the images, the asymptomatic carriers were recognized as normal controls. In the subsequent data analysis, carriers were combined with the normal subject group. Generalized estimating equations were performed to compare variations in the quantitative data among symptomatic carriers, asymptomatic carriers, and normal subjects. κ statistics were applied to determine interobserver reproducibility by estimating agreement between the two observers. For the comparison of mean RNFL visibility scores between the two observers, the Standard *t*-test was used, and the one-way analysis of variance with *post-hoc* Tukey analysis was used for the same comparison between MC and CFP. The sensitivity and specificity values with corresponding 95% CI were calculated to evaluate the diagnostic performance of each imaging approach for the detection of never fiber bundle defects. McNemar's test was used to compare the diagnostic performance of each approach. The data were analyzed using IBM SPSS Statistics 25 software (IBM Corp). A value of *P* < 0.05 was considered statistically significant.

## Results

A total of 46 symptomatic carriers (17 women and 29 men, mean age of 24.14 years) with the *G11778A* mutation, eight asymptomatic carriers (7 women and 1 man, mean age of 36.1 years), and 20 normal subjects (5 women and 15 men, mean age of 39.8 years) were recruited. Eleven eyes were excluded due to poor imaging quality. There are baseline characteristics in Table 1. Twenty eyes were in the subacute stage, with an average disease duration of 3.69 months. Eighteen eyes were in the dynamic stage, with an average disease duration of 9.32 months. Forty-four eyes were in the chronic stage, with an average disease duration of 58.64 months.

**Table 1:** Baseline characteristics of participants.

	**Symptomatic carriers**			**Asymptomatic carriers**	**Normal subjects**
	**Subacute**	**Dynamic**	**Chronic**		
Age, y		24.14		36.1	39.8
Sex, Male/Female		54/28		2/14	29/10
Number (eyes)	20	18	44	16	39
Superior pRNFL thickness (μm)	156.15 ± 30.51	88.17 ± 10.78	62.84 ± 19.57	132.56 ± 35.08	131.79 ± 14.37
Nasal pRNFL thickness (μm)	70.80 ± 16.08	46.83 ± 12.45	31.82 ± 15.76	61.31 ± 18.50	55.50 ± 12.31
Inferior pRNFL thickness (μm)	147.65 ± 17.20	79.67 ± 11.25	60.50 ± 20.39	131.94 ± 39.27	124.57 ± 17.73
Temporal pRNFL thickness (μm)	63.85 ± 27.80	29.33 ± 1.51	29.56 ± 6.64	75.44 ± 22.32	92.79 ± 19.08
Mean pRNFL thickness (μm)	119.70 ± 15.21	60.83 ± 5.71	46.20 ± 13.58	100.38 ± 26.35	101.29 ± 7.75

RNFL, retinal nerve fiber layer.

Age, sex, spherical equivalent, and IOP values were not significantly different between these three groups; however, logMAR VA, visual field indices (VFIs), mean deviation (MD), and pRNFL thickness were significantly worse in LHON (*P* < 0.05).

### RNFL visibility score and interobserver agreement

Retinal nerve fiber layer visibility scores were assigned based on the earlier noted 4-point system. BR (2.71 ± 0.55) showed the best mean RNFL visibility score, followed by BG (2.69 ± 0.52), GR (2.69 ± 0.56), standard MC (2.04 ± 0.79), CFP (1.80 ± 0.82), and IR (0.45 ± 0.59) (Table 2). Short-wavelength light images revealed the never fiber bundle defects significantly more clearly than CFP and IR images, as well as a higher average RNFL visibility score (all *P* < 0.05). Interobserver agreement (κ) on the degree and location of never fiber bundle defects and clinical stages of LHON was substantial for CFP, standard MC, GR, BR, and BG (all κ > 0.620) (Table 2). Interobserver agreement (κ) regarding the evaluation of the never fiber bundle defects in the temporal region was generally superior to that in other areas. The average agreement on the detection of never fiber bundle defects was lowest for IR (κ = 0.545) and highest for GR (κ = 0.774) and BR (0.776). In addition, we compared the consistency of clinical staging judgments based on never fiber bundle defects in each independent image and pRNFL thickness. BG (κ = 0.893) showed the best consistency, followed by BR (κ = 0.861), GR (κ = 0.833), standard MC (κ = 0.701), CFP (κ = 0.625), and IR (κ = 0.109) (Table 2).

**Table 2:** Retinal nerve fiber layer visibility score and interobserver agreement (κ) regarding the degree of retinal nerve fiber layer defects and clinical stages of LHON.

	**RNFL visibility score**	**Interobserver agreement (**κ**)**					**Clinical stages agreement (κ)**
		**Superior RNFL**	**Nasal RNFL**	**Inferior RNFL**	**Temporal RNFL**	**Clinical stages**	
CFP	1.80 ± 0.82	0.769	0.620	0.770	0.835	0.751	0.625
Standard MC	2.04 ± 0.79	0.695	0.672	0.752	0.916	0.902	0.701
IR	0.45 ± 0.59	0.570	0.550	0.521	0.540	0.496	0.109
GR	2.69 ± 0.56	0.729	0.661	0.748	0.957	0.884	0.833
BR	2.71 ± 0.55	0.729	0.641	0.803	0.930	0.852	0.861
BG	2.69 ± 0.52	0.708	0.635	0.769	0.890	0.841	0.893

RNFL, retinal nerve fiber layer; CFP, color fundus photography; MC, multicolor; IR, infrared reflectance; GR, green reflectance; BR, blue reflectance; BG, green-blue-enhanced imaging.

### Performance of multicolor images for the identification of never fiber bundle defects on the PMB area

In this study, we assessed whether RNFL in PMB could serve as a distinguishing factor between patients with LHON and normal subjects. The sensitivity of LHON detection using CFP was 82.75%, with a specificity of 95.45%, PPV of 88.89%, NPV of 92.64%, and Youden's index of 78.21%. The sensitivity of LHON detection using standard MC was 89.74%, with a specificity of 94.74%, PPV of 89.74%, NPV of 94.74%, and Youden's index of 84.48%. The sensitivity of LHON detection using IR was 45.45%, with a specificity of 100.00%, PPV of 100.00%, NPV of 66.67%, and Youden's index of 45.45%. The sensitivity of LHON detection using GR was 94.87%, with a specificity of 96.05%, PPV of 92.50%, NPV of 97.33%, and Youden's index of 90.92%. The sensitivity of LHON detection using BR was 92.31%, with a specificity of 97.33%, PPV of 94.74%, NPV of 96.05%, and Youden's index of 89.64%. The sensitivity of LHON detection using BG was 92.31%, with a specificity of 98.68%, PPV of 97.30%, NPV of 96.15%, and Youden's index of 90.99%. The Youden's indices of GR, BR, and BG all exceeded 89.00% (Table 3).

**Table 3:** Sensitivity and specificity of multicolor images and color fundus photography in detecting Leber hereditary optic neuropathy.

	**Sensitivity (%)**	**Specificity (%)**	**PPV (%)**	**NVP (%)**	**Youden indices (%)**
CFP	82.75	95.45	88.89	92.64	78.21
Standard MC	89.74	94.74	89.74	94.74	84.48
IR	45.45	100.00	100.00	66.67	45.45
GR	94.87	96.05	92.50	97.33	90.92
BR	92.31	97.33	94.74	96.05	89.64
BG	92.31	98.68	97.30	96.15	90.99

PPV, positive predictive value; NPV, negative predictive value; CFP, color fundus photography; MC, multicolor; IR, infrared reflectance; GR, green reflectance; BR, blue reflectance; BG, green-blue-enhanced imaging.

### Sensitivity and specificity in detecting clinical stages of LHON

**Figure 1:**
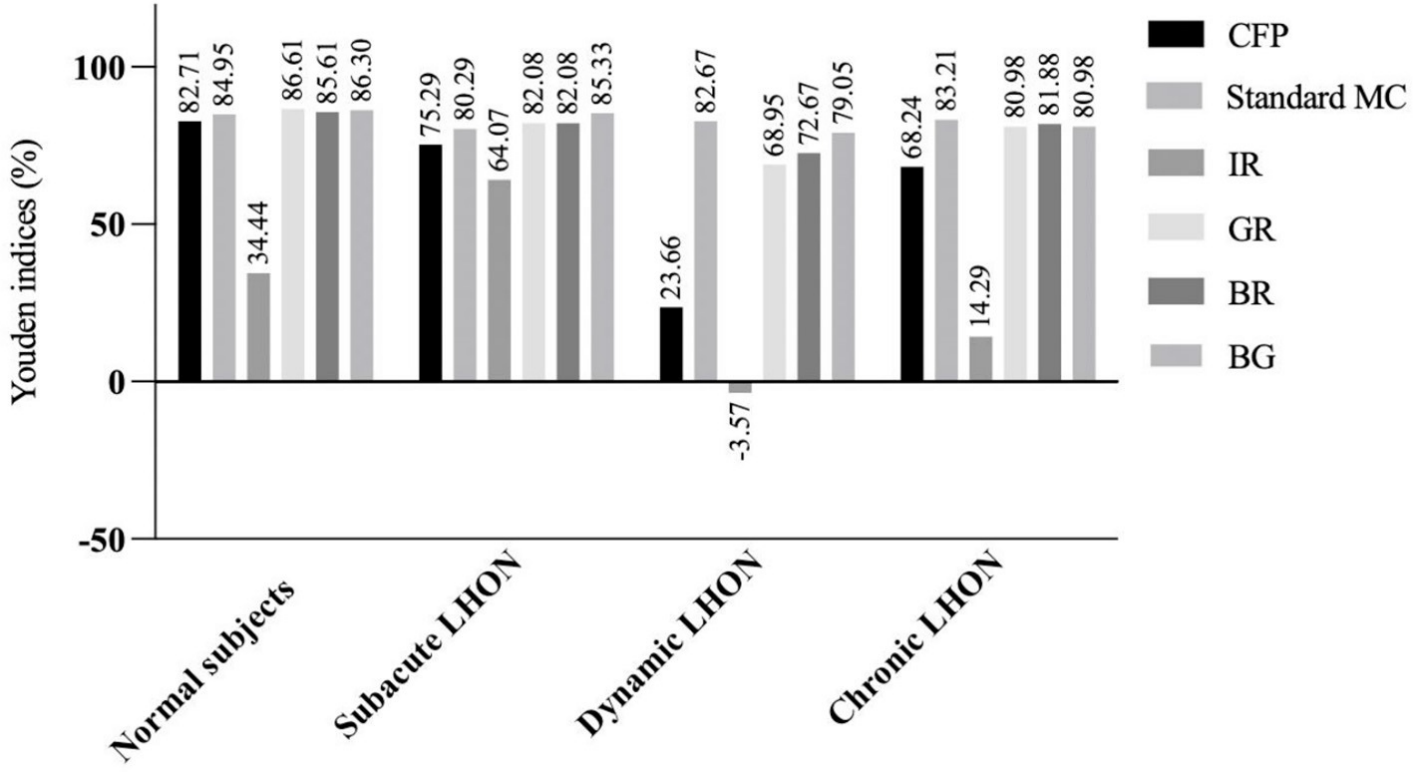
Youden's indices (%) of multicolor images and color fundus photography in detecting clinical stages of Leber hereditary optic neuropathy. LHON, Leber hereditary optic neuropathy; CFP, color fundus photography; MC, multicolor; IR, infrared reflectance; GR, green reflectance; BR, blue reflectance; BG, green-blue-enhanced imaging.

We found that the Youden's indices of GR and BG for the detection of normal subjects exceeded 86% (Figure 1). Standard MC, GR, BR, and BG exhibited the highest Youden's indices (>80%) for the detection of subacute LHON. Standard MC had the highest Youden's index (82.67%) for the detection of dynamic LHON. GR, BR, and BG all had Youden's indices above 68% for the detection of dynamic LHON. Standard MC had the highest Youden's index (83.21%) for the detection of chronic LHON. GR, BR, and BG had Youden's indices above 80.00% for the detection of chronic LHON.

### Representative cases of LHON based on multicolor images and color fundus photography of patient eyes

**Figure 2:**
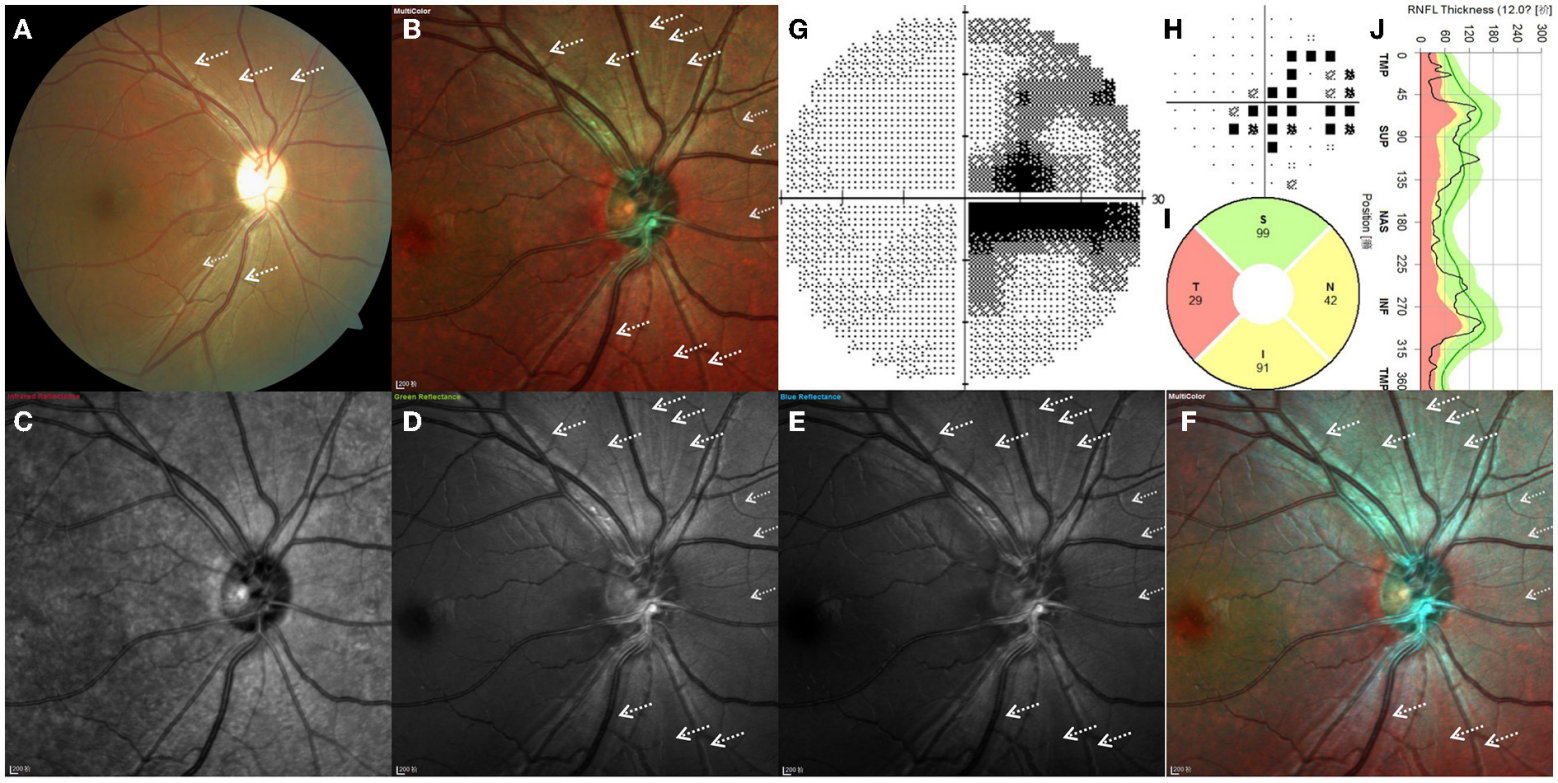
**(A–J)** Right eye of a 28.4-year-old Leber hereditary optic neuropathy patient with the *G11778A* mutation. White arrows indicate the positions of retinal nerve fiber layer defects.

Figure 2 depicts the right eye of a 28.4-year-old man with a 15-month history of sudden vision loss in both eyes. He was diagnosed with LHON with the *G11778A* mutation. CFP, standard MC, GR, BR, and BG images generally enable high RNFL visibility, while the fibers are barely visible in IR images (Figure 2). By CFP, the optic disc was pale, and the reflection was overly strong, resulting in overexposure (Figure 2A). The retinal never fibers were gray and radiated out from the optic disc. In the temporal region, fibers are hardly visible, consistent with the typical characteristics of patients with LHON. In other quadrants, fiber defects exhibited a fissure shape and were of inconsistent sizes. Standard MC, GR, BR, and BG images enabled clear visual delineation of the margins of RNFL defects, in contrast to the indistinguishable margins associated with conventional photography (Figures 2B–F). The scotoma on the VF examination corresponds to wide-fissured fiber loss in CFP and MC (Figures 2G,H). By OCT, the average pRNFL thickness in the temporal region was found to be significantly different. In other quadrants, pRNFL pseudoedema disappeared and atrophy gradually became apparent, especially in the inferior and nasal regions (Figures 2I,J).

### Representative cases of a normal subject, an asymptomatic carrier, and patients with LHON at different clinical stages

**Figure 3:**
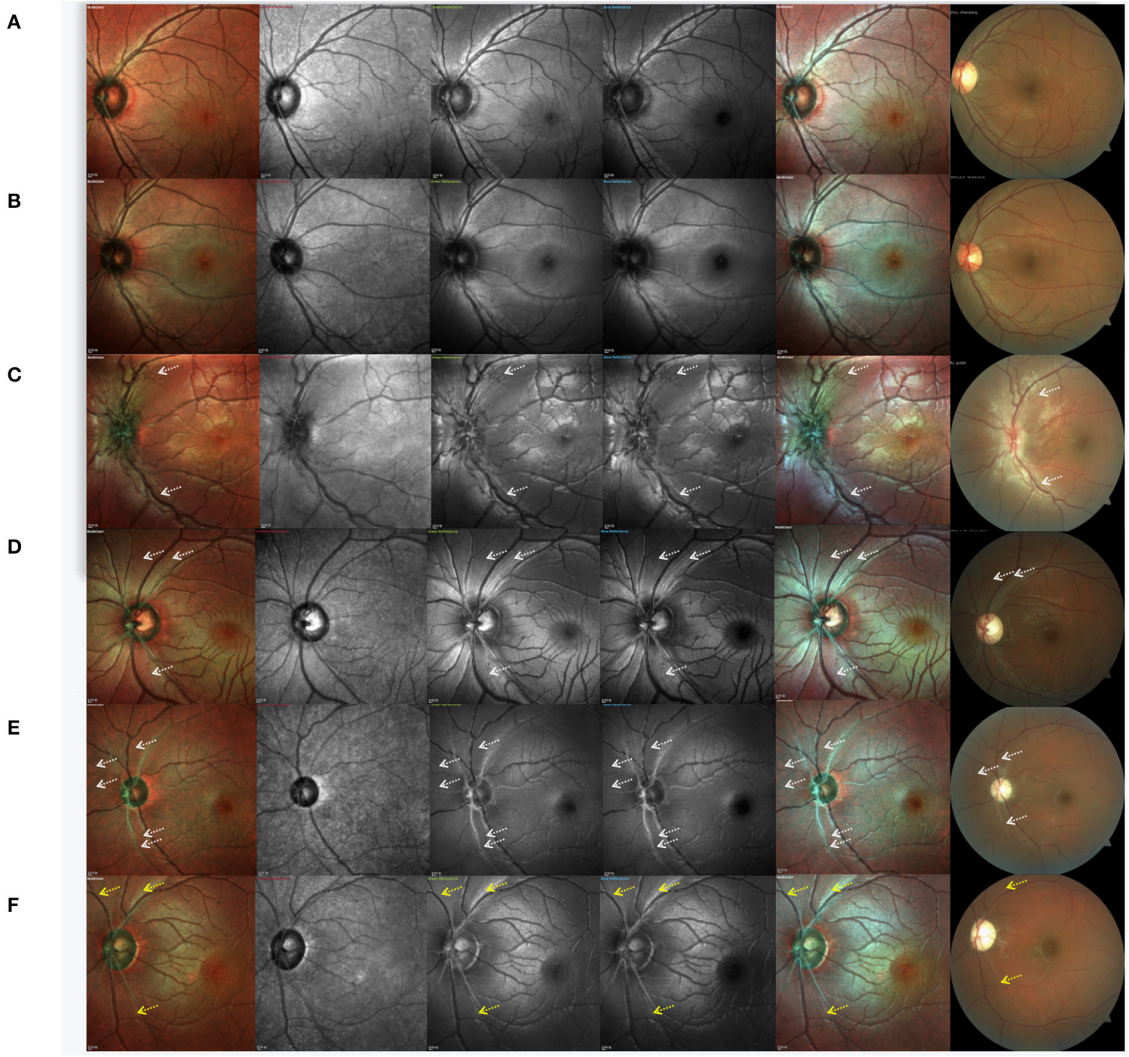
Cases of a normal subject, asymptomatic carrier and Leber hereditary optic neuropathy patients at different clinical stages. **(A)** normal subject, **(B)** asymptomatic mutation carrier, **(C)** LHON at the subacute stage, **(D)** LHON at the dynamic stage, and **(E,F)** LHON at the chronic stage. White arrows indicate the positions of retinal nerve fiber layer defects, and yellow arrows indicate remnants of the retinal nerve fiber layer.

Case 1. Normal subject: Standard MC, GR, BR, and BG images revealed a pattern of fine, subtle, and healthy RNFL striation, which was more distinct by GR, BR, and BG imaging than by standard MC (Figure 3A).

Case 2. Asymptomatic mutation carrier: By CFP, the optic disc of asymptomatic mutation carriers was shown to be hyperemic. RNFL swelling can also be detected by OCT as increased RNFL thickness in the inferior quadrant. However, no significant never fiber bundle defects were found by MC or CFP (Figure 3B). In IR images, RNFL was faintly visible.

Case 3. LHON at the subacute stage (<6 months from onset): CFP, standard MC, GR, BR, and BG imaging generally indicated an almost complete loss of retinal never fibers, and no white fibers could be seen in the temporal region. We also observed that subacute patients with LHON had retinal nerve fibers that were more distinct and of larger diameter (more swollen) than those of normal subjects, except in the temporal quadrant. Even in IR images, swollen fibers were faintly visible. The capillaries around the optic disc were significantly dilated and tortuous. Visible hyperemia of the optic disc could also be seen by CFP (Figure 3C).

Case 4. LHON at the dynamic stage (6–12 months): In addition to the loss of white fibers in the temporal region, never fiber bundle defects could be found in other quadrants. Contrasting never fiber bundle defects were more distinct by standard MC, GR, BR, and BG imaging compared to CFP. Never fiber bundle defects appeared as small fissures connected to the optic disc. By CFP, the optic disc was pale (Figure 3D).

Case 5. LHON at the chronic stage (>12 months): CFP, standard MC, GR, BR, and BG imaging generally revealed that RNFL defects across all quadrants appeared as broader fissures connected to the optic disc, and the number of fissures was significantly higher than what was observed in the dynamic stage (Figure 3E). In LHON patients with longer disease duration, RNFL was almost invisible in the entire optic disc. Along the superior temporal and inferior temporal retinal vessels, some remnant fibers were visible. By CFP, the optic disc was pale (Figure 3F).

## Discussion

MC imaging is a high-contrast imaging modality that uses three different laser sources to visualize the ocular fundus. Different wavelengths of lasers enable the collection of information originating from different retinal structures, resulting in increased detail and contrast (13). Our results showed good interobserver agreement regarding never fiber bundle defects and performance of CFP, standard MC, GR, BR, and BG images relative to IR images. The low interobserver agreement of IR corresponds to low RNFL visibility. Infrared wavelength lasers are known to be largely transmitted through the retina and are ideal for imaging changes in the retinal pigment epithelium and choroid, although not for imaging of RNFL (13). A precise pattern of RNFL defects had been substantiated by OCT (14) and histopathology studies, (15) characterized by the preferential involvement of small axons. A necessary density of small susceptible axons initiates a locally cascading destructive event, engulfing neighbor cells regardless of size in a “common reservoir” phenomenon. Then, the affected sectors of LHON optic nerves are completely devoid of axons, with losses extending beyond the smallest and most susceptible fibers. The presence of RNFL defects in the form of retinal nerve fiber bundles allows us to intuitively judge RNFL defects and retention using fundus photography. The inferotemporal aspect of the optic nerve fiber, PMB, contains the smallest axons by average diameter (15, 16). These small axons are the most sensitive to energy depletion, as they cope less efficiently through compensatory mechanisms (2). Upon LHON onset, PMB becomes preferentially involved, and the VF examination revealed a central scotoma. In this study, we employed PMB involvement as a factor to distinguish patients with LHON from normal subjects. The Youden's indexes of CFP, standard MC, GR, BR, and BG in the present study generally indicated good detection of never fiber bundle defects in patients with PMB/LHON, with BG and GR exhibiting obvious advantages (Youden's index >90%). In addition, the present study determined that GR, BR, and BG were associated with higher RNFL visibility scores than CFP and IR. RNFL in the temporal region had the highest interobserver agreement. LHON is representative disease with preferential fibers defects in the PMB area, for example, autosomal dominant optic atrophy (ADOA) and toxic optic neuropathy. Although patients with other optic neuropathies, such as glaucoma, anterior ischemic optic neuropathy, and optic neuritis were not included in our study and MC alone is not diagnostic of LHON, high-quality imaging of small PMB axons by MC can also enable the distinction of suspicion of LHON from others, especially optic neuritis. The complete defects of never fiber in the PMB area, in combination with the population and ophthalmologic characteristics, will enable targeted genetic examination of patients and significantly reduce missed diagnoses and shorten the diagnostic cycle.

Previous studies have demonstrated a relationship between the clinical stages of LHON and pRNFL thickness (14). A certain pattern of pRNFL involvement in LHON has been described; first, the temporal pRNFL thins, followed by thinning of the inferior and superior quadrants, and finally, of the nasal pRNFL (2, 14). Based on the characteristics of nerve fiber bundle loss in patients with LHON visualization of the ocular fundus and description of fiber subregions may provide new insights into the clinical stages of LHON. The resolution of each scan in MC-cSLO is 3.5 μm/pixel; thus, this approach produces clearer and more detailed images than CFP. Several studies have reported the usefulness of this technique for the detection of RNFL defects in retinal diseases (10, 11, 17). The present study found that the stage of LHON judged by the proportion of fiber bundle defects in standard MC, GR, BR, and GB was highly consistent with the course inferred by pRNFL thickness. In comparison to CFP and IR, standard MC, GR, BR, and BG imaging performed better in detecting clinical stages of LHON. This difference was especially significant when assessing patients with LHON in the subacute and chronic stages. Standard MC has obvious advantages in judging patients across all stages, although standard MC had only middling performance regarding RNFL visibility. The degree of background pigmentation and remaining RNFL thickness has been reported as the two major factors contributing to good RNFL visibility (18). Despite the reliable grading of RNFL visibility by standard MC, it ranked lowest among MC approaches and was probably affected by the low-quality portions of each wavelength image. However, standard MC was more reliable in detecting clinical stages of LHON. This may be due to the composite characteristics of standard MC. GR and BR enable the detection of a delicate texture of RNFL and remnant fibers, and variations in the relative signals detected from each laser wavelength are accentuated at RNFL defect margins. In addition, IR allows clearer visualization of the retinal pigment epithelium (RPE) and choroid when assessing never fiber bundle defects, increasing background contrast for white reflective RNFL and, thus, firmly differentiating the defective area from the unaffected region.

There are some limitations to this study. First, we used 30° MC imaging, which limited the evaluation of RNFL in the nasal area. However, because RNFL defects in the nasal area are not dominant, we considered this approach sufficient for evaluating LHON structural defects and detecting clinical stages. Second, we summarized asymptomatic carriers to normal controls for data analysis. To characterize patients with LHON at all stages, including the asymptomatic, we included LHON asymptomatic carriers in our study. Carriers were also reported to have mild thickening of the pRNFL thickness (19). However, when masked to read images, all the asymptomatic carriers were identified as normal controls, and there were no significant fiber bundle differences between the MC images of asymptomatic carriers and normal controls. The inclusion of carrier data in normal controls will only possibly reduce the differential significance without compromising our findings. Third, all enrolled patients were Asian, so generalizing our results to other ethnicities may not be appropriate. Because Asians have thick RNFL and hyperpigmented retinas, reflection from the thicker RNFL is prominent, and reflection from the deeper hyperpigmented retina is less visible. Moreover, the color of MC images can be altered by different diopter and exposure parameters. Therefore, ophthalmologists should pay extra attention when distinguishing the appearance of pathologic changes arising from artifacts. Finally, these imaging modalities are nonquantitative.

In conclusion, MC imaging, especially GR, BR, and BG, enables good RNFL visibility compared to CFP and IR. It also exhibited good interobserver agreement regarding never fiber bundle defects and performance of CFP, standard MC, GR, BR, and BG images relative to IR. BG and GR imaging were generally good for the detection of fiber bundle defects in the PMB area. For detecting clinical stages of LHON, MC, GR, BR, and BG approaches generally performed better than CFP or IR. These differences were especially significant when judging patients with LHON in the subacute and chronic stages. Standard MC exhibited obvious advantages in the assessment of patients with LHON across all stages, despite a mediocre RNFL visibility score. Therefore, MC can be beneficial in the detection of suspected patients with LHON, and defects of fiber bundle on fundus photography may also benefit on the staging of LHON.

## Data availability statement

The original contributions presented in the study are included in the article/supplementary material, further inquiries can be directed to the corresponding author/s.

## Ethics statement

The studies involving human participants were reviewed and approved by the Clinical Research Ethics Committee of Renmin Hospital of Wuhan University (WDRY2021-K018). Written informed consent to participate in this study was provided by the participants' legal guardian/next of kin.

## Author contributions

Conceptualization and data curation: YC and LH. Methodology: YC, CC, and WW. Formal analysis: YC, CC, QM, and JY. Investigation: YC, QM, WW, and JY. Project administration: YC, CC, and JY. Resources: JY. Software and writing—original draft: YC. Supervision: CC and JY. Validation: YC, LH, and CC. Visualization: YC and CC. Writing—review and editing: LH and CC. All authors have read and agreed to the published version of the manuscript.

## Funding

This work was supported by the National Natural Science Foundation of China (Grant No. 82101115) and the Wuhan University Independent Innovation Fund Youth Project (Grant No. 2042021kf0094). The funders had no role in the design of the study, in the collection, analyses, or interpretation of data; in the writing of the manuscript; or in the decision to publish the results.

## Conflict of interest

The authors declare that the research was conducted in the absence of any commercial or financial relationships that could be construed as a potential conflict of interest.

## Publisher's note

All claims expressed in this article are solely those of the authors and do not necessarily represent those of their affiliated organizations, or those of the publisher, the editors and the reviewers. Any product that may be evaluated in this article, or claim that may be made by its manufacturer, is not guaranteed or endorsed by the publisher.
